# High-resolution, in vivo multimodal photoacoustic microscopy, optical coherence tomography, and fluorescence microscopy imaging of rabbit retinal neovascularization

**DOI:** 10.1038/s41377-018-0093-y

**Published:** 2018-12-05

**Authors:** Wei Zhang, Yanxiu Li, Van Phuc Nguyen, Ziyi Huang, Zhipeng Liu, Xueding Wang, Yannis M. Paulus

**Affiliations:** 1 Department of Biomedical Engineering, University of Michigan, Ann Arbor, MI 48105 USA; 2 Institution of Biomedical Engineering, Chinese Academy of Medical Sciences and Peking Union Medical College, Tianjin, 300192 China; 3 Department of Ophthalmology and Visual Sciences, University of Michigan, Ann Arbor, MI 48105 USA; 4 Department of Ophthalmology, Xiangya Hospital, Central South University, Changsha, Hunan 410008 China; 5 Department of Radiology, University of Michigan, Ann Arbor, MI 48105 USA

## Abstract

Photoacoustic microscopy (PAM) is an emerging imaging technology that can non-invasively visualize ocular structures in animal eyes. This report describes an integrated multimodality imaging system that combines PAM, optical coherence tomography (OCT), and fluorescence microscopy (FM) to evaluate angiogenesis in larger animal eyes. High-resolution in vivo imaging was performed in live rabbit eyes with vascular endothelial growth factor (VEGF)-induced retinal neovascularization (RNV). The results demonstrate that our multimodality imaging system can non-invasively visualize RNV in both albino and pigmented rabbits to determine retinal pathology using PAM and OCT and verify the leakage of neovascularization using FM and fluorescein dye. This work presents high-resolution visualization of angiogenesis in rabbits using a multimodality PAM, OCT, and FM system and may represent a major step toward the clinical translation of the technology.

## Imaging eye disease

A multimodal imaging system which can visualize abnormal new blood vessels in the retina and choroid could be a useful future tool for diagnosing and monitoring eye diseases. Retinal neovascularization can lead to significant loss of vision through diseases such as age-related macular degeneration, diabetic retinopathy, sickle cell retinopathy, and retinal vein occlusions. This manuscript describes a novel high-resolution imaging system that combines optical coherence tomography (OCT), photoacoustic microscopy (PAM), and fluorescence microscopy (FM) and demonstrates that it can visualize neovascularization in the eyes of living rabbits.

## Introduction

Retinal neovascularization (RNV) represents a major cause of vision loss and blindness and is a common complication of numerous retinal diseases, including proliferative diabetic retinopathy, retinopathy of prematurity, sickle cell retinopathy, and retinal vein occlusions^1–5^. Current imaging methods to diagnose RNV include fluorescein angiography (FA), indocyanine green angiography (ICGA), optical coherence tomography (OCT), and OCT angiography (OCTA)^6^. FA permits visualization of retinal circulation in detail, but its role in studying choroidal circulation is limited due to free permeation of fluorescein in choroidal vessels. Using a larger fluorescent molecule with 98% binding to plasma, ICGA can reveal choroidal circulation, but the images are often difficult to interpret. In addition, both FA and ICGA are invasive and require intravenous injection of exogenous dyes that may cause complications in up to 10% of patients, including nausea, emesis, anaphylactic reactions, and even death^7^. OCT and OCTA provide anatomic information with high resolution and excellent penetration depth^8,9^. However, OCTA does not exhibit leakage, provides limited visualization of microaneurysms, and has a restricted field of view, often with artifacts. This suggests that a multimodality optical imaging approach that can combine the advantages of OCT with additional functional and molecular information would be very beneficial in the field of ophthalmology^10^.

As a hybrid biomedical imaging method, photoacoustic microscopy (PAM) has the unique capability to non-invasively explore the optical absorption properties in biological tissues with high spatial and temporal resolution^11^. PAM has been widely used in preclinical research, including studies of tumor^12^, brain^13,14^, bone^15^, liver^16^, and joint tissues^17^. A nanosecond-pulse-duration laser is used to achieve localized thermoelastic expansion of the target tissue. This causes acoustic waves to be emitted from the target area, which can be detected with ultrasound transducers and reconstructed to obtain photoacoustic imaging with high contrast and resolution^18^. Due to the optical transparency, the eye and retina are considered very suitable for the application of PAM^19–22^.

**Fig. 1:**
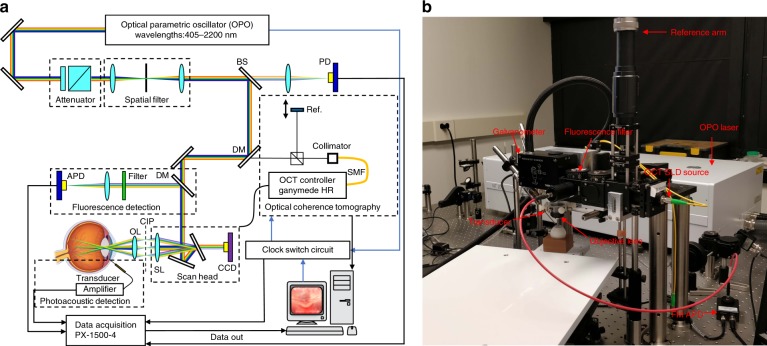
Integrated multimodality retinal imaging system. **a** Schematic; **b** photograph of the multimodality retinal and choroidal imaging system. (OPO optical parametric oscillator; SLD superluminescent diode; BS beam splitter; DM dichroic mirror; SL scan lens; OL ophthalmic lens; CIP conjugate image planes)

A multimodality imaging system with integrated OCT and PAM used to evaluate normal rabbit eyes has recently been described^23,24^. Whereas PAM has been employed in corneal neovascularization^25^ and tumor microvasculature^26^, high-resolution PAM retinal imaging, particularly in large animal eyes, faces numerous technical challenges, including ultrasound signal attenuation with distance, particularly for high-frequency components, corneal dehydration, and optical aberrations, and thus most studies have been limited to mice and rats. This study performs the first PAM evaluation of RNV in vivo in large animal eyes. RNV was induced in living albino and pigmented rabbits to test the feasibility of the integrated multimodality PAM, OCT, and fluorescence microscopy (FM) system illustrated in Fig. 1.

## Results

### RNV model

**Fig. 2:**
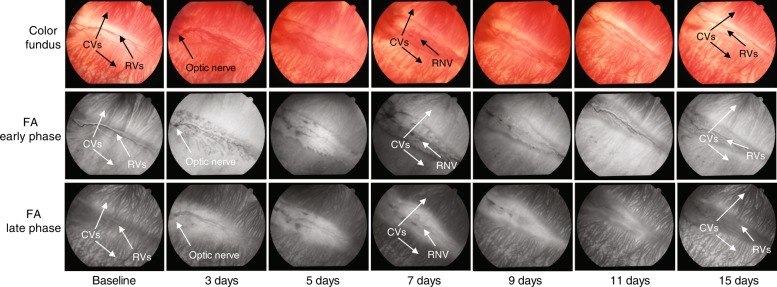
Dynamic changes of VEGF-induced rabbit RNV. Color fundus images and fundus FA images of VEGF-induced rabbit RNV in albino rabbit at different time points.

To evaluate rabbit RNV, baseline imaging of normal rabbit retinal vasculature was first performed in normal albino and pigmented rabbits before vascular endothelial growth factor (VEGF) injection. Imaging was performed at 1, 3, 5, 7, 9, 11, 13, 15, 20, 25, and 30 days after VEGF injection and demonstrated that the VEGF-induced RNV reached its peak at 7 days. Fundus and FA images of VEGF-induced RNV at different time points are shown in Fig. 2. Based on this peak of 7 days in the VEGF RNV model, 7 days was selected as the time point for subsequent imaging of RNV using the multimodality PAM, OCT, and FM system.

### Normal retinal vessels

**Fig. 3:**
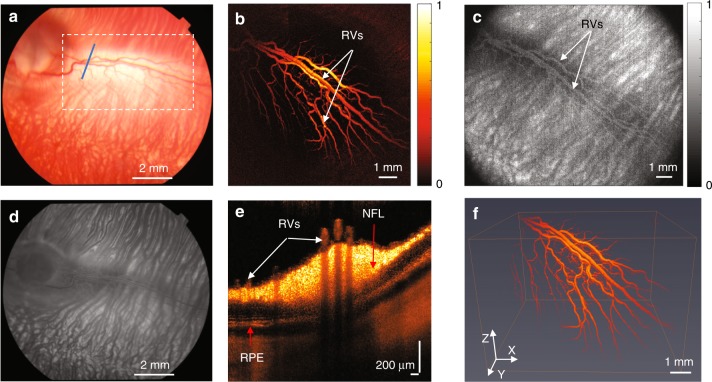
Normal retinal vessels in albino rabbits. **a** Color fundus image; **b** 2D PAM image indicated by white dashed box in **a**; **c** FM image; **d** fundus FA image; **e** OCT B-scan image indicated by blue line in **a**; **f** 3D reconstruction of PAM image. (White arrows indicate normal retinal vessels; NFL nerve fiber layer; RPE retinal pigment epithelium)

Fundus images of normal retinal vessels in albino rabbits were obtained using a Topcon TRC-50EX (Topcon Corporation, Tokyo, Japan) (Fig. 3a). Figure 3d is the corresponding FA image acquired right after the fundus image, which was used to demonstrate the retinal vasculature and demonstrates progressive hyperfluorescence with blurred margins consistent with leakage from neovascularization. In Fig. 3b, the PAM image clearly shows the retinal vasculature in the targeted area. Both the main vessels and vascular branches in the rabbit retina can be easily distinguished using PAM. FM images were acquired after PAM. Fluorescein sodium was used as a dye to obtain the FM image with 480-nm incident light. After the laser finished scanning the area, the fluorescence signals obtained at each point were combined to reconstruct an FM image, which is shown in Fig. 3c. Although, we can distinguish the retinal vessels in the FM image, the strong background signal from dense choroidal vessels deceased the contrast of the image. Different layers of retina are evident in the OCT B-scan image shown in Fig. 3e. The cross-section of the main vessels and the vascular branches of the retinal vasculature are indicated by white arrows. After removing the signal from the choroidal layer, the photoacoustic signal acquired from retinal vessels was reconstructed into 3D imaging (see Video. 1) using Amira 6.4 (FEI, Hillsboro, OR, USA). The 3D structure of the normal retinal vessels is shown in Fig. 3f.

**Fig. 4:**
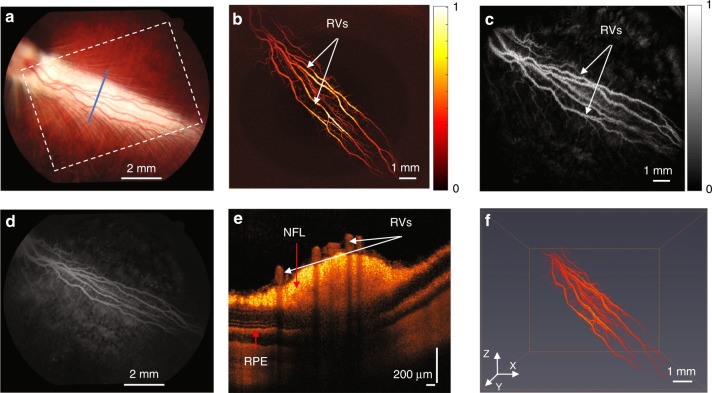
Normal retinal vessels in pigmented rabbits. **a** Color fundus image; **b** 2D PAM image indicated by white dashed box in **a**; **c** FM image; **d** fundus FA image; **e** OCT B-scan image indicated by blue line in **a**; **f** 3D reconstruction of PAM image. (White arrows indicate normal retinal vessels; NFL nerve fiber layer; RPE retinal pigment epithelium)

The same images were acquired with pigmented rabbits in vivo to determine whether melanin would impede the acquisition of high-quality images. Melanin has a broad absorption peak, and its proximity to the vasculature previously caused difficulty in performing PAM imaging. In addition, melanin from the retinal pigment epithelium (RPE) overlies the choroid and choriocapillaris and thus could possibly block the diffuse choroidal hyperfluorescence since fluorescein is freely permeable in choroidal vessels. As shown in Fig. 4a, pigmented rabbits exhibit a dark fundus with a white medullary ray. The corresponding FA image for the same area, shown in Fig. 4d, demonstrates improved visualization of retinal vasculature due to blockage of the diffuse choroidal hyperfluorescence. The PAM image shows that the retinal vasculature is visible more clearly than for the albino rabbit (Fig. 3b). The FM image also has improved contrast compared to the albino rabbit due to less choroidal hyperfluorescence, which allows improved visualization of the microvasculature (Fig. 4c). The OCT B-scan image of the cross-section at the position of the blue line in Fig. 4a is shown in Fig. 4e. In addition, the cross-section of the retinal vessels shown in the OCT image corresponds to the photoacoustic image. As shown in Fig. 4f, the photoacoustic signal from the retinal vasculature was reconstructed in a 3D image (see Video. 2) to show its spatial distribution. This demonstrates not only that high-quality PAM, OCT, and FM of the retinal vasculature is possible with pigmented rabbits but also that the melanin improves the image quality of all 3 modalities.

### RNV

**Fig. 5:**
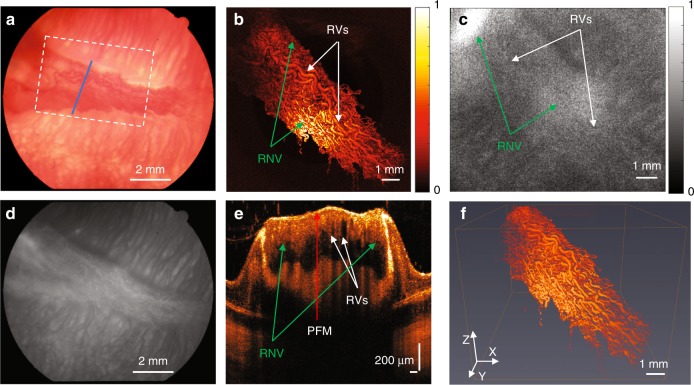
RNV in albino rabbits. **a** Color fundus image; **b** 2D PAM image indicated by white dashed box in **a**; **c** FM image; **d** fundus FA image; **e** OCT B-scan image indicated by blue line in **a**; **f** 3D reconstruction of PAM image. (White arrows indicate normal retinal vessels; green arrows indicate RNV; PFM preretinal fibrovascular membrane)

The multimodality images of albino rabbits with the RNV model were obtained 7 days after VEGF injection. According to the fundus image in Fig. 5a, the large vessels have a significant increase in tortuosity and numerous new tortuous angiogenic vessels have now grown over the retina, consistent with RNV. Whereas the myelinated nerve fibers of the medullary ray previously caused a white streak across the fundus photograph, a network of small, irregular neovascular vessels now covers it, changing the white streak to red. The FA image in Fig. 5d demonstrates significant late progressive hyperfluorescence with blurred margins, consistent with extensive leakage from neovascularization. From the PAM image shown in Fig. 5b, the tortuous normal retinal vessels can still be distinguished, but the image now also reveals an irregular network of small, irregular neovascularization (indicated by green arrows) around the normal vasculature. The normal, organized retinal vasculature becomes a dense bundle of tortuous blood vessels, indicating that the normal retinal blood vessels are surrounded by RNV. Compared with the normal retinal vasculature without VEGF injection with clearly demarcated vessels, RNV, in contrast, leads to difficulty in distinguishing the blood vessel branches and main vessel. For the FM image shown in Fig. 5c, there is significant diffuse leakage from the extensive neovascularization, which makes discerning vascular structures very challenging. The leakage of fluorescein sodium from RNV induces a strong diffuse fluorescence signal through the field of view. In addition, fluorescence from the choroidal layer also causes diffuse background fluorescence. As a result of this diffuse leakage from both vascular supplies, the contrast between retinal vessels and the background was too low to be detected by the digitizer. Although the preretinal fibrovascular tissue covers the retinal vasculature, which will block most of the OCT light, the corresponding irregular structures were shown clearly in the OCT image in Fig. 5e as a thick network of blood vessels on the inner retinal surface. Normal vasculature has thicker, more mature blood vessel walls than RNV, and thus, normal vasculature has more shadowing below it than RNV, as revealed by the OCT image. The vascular structures with less shadowing below them indicate RNV. Via comparison with the normal retinal vessels in Fig. 3b, e, the structures that have strong photoacoustic signals and thinner blood vessel walls should represent the neovascularization. To eliminate the influence from the choroidal layer, the photoacoustic signal from the retinal layer was exacted from the raw data to perform a 3D reconstruction (see Video. 3). As shown in Fig. 5f, the distribution and the difference between normal retinal vessels and the RNV are shown in the 3D image reconstruction.

**Fig. 6:**
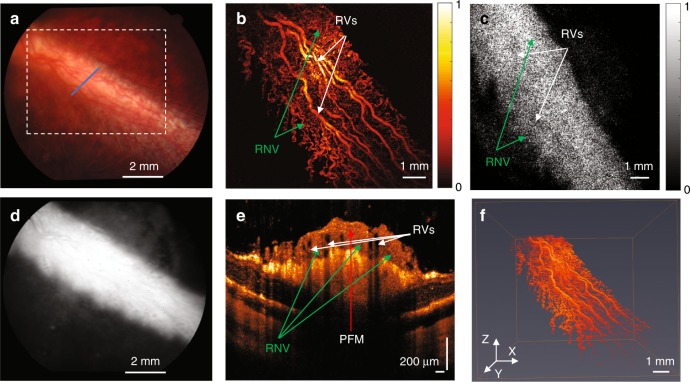
RNV in pigmented rabbits. **a** Color fundus image; **b** 2D PAM image indicated by white dashed box in **a**; **c** FM image; **d** fundus FA image; **e** OCT B-scan image indicated by blue line in **a**; **f** 3D reconstruction of PAM image. (White arrows indicate normal retinal vessels; green arrows indicate RNV; PFM preretinal fibrovascular membrane)

To ensure clinical translatability given the significant melanin present in most human eyes, a similar study was performed with pigmented rabbits. The multimodality images were also acquired for pigmented rabbits 7 days after VEGF injection to induce the RNV. Compared with the images acquired before RNV induction, the fundus photographs and FA images show that the RNV induced by VEGF and the leakage of fluorescein dye from the RNV are clearly discernable (Fig. 6a, d). Similar to the albino rabbits, pigmented rabbits have a significant increase in vascular tortuosity, which can be distinguished in the PAM image shown in Fig. 6b. Some strong photoacoustic signals around the normal retinal vessels can also be detected; these represent small, irregular neovascularization (indicated by green arrows). Although a dense network of tortuous neovascularization grew around the retinal blood vessel layer, PAM still can distinguish each individual vessel. These OCT images also reveal a network of blood vessels on the inner retinal surface, shown in Fig. 6e. FM demonstrates significant progressive hyperfluorescence with blurred margins, consistent with leakage of fluorescein dye, shown in Fig. 6c, which is more easily visualized in pigmented rabbits than in albino rabbits due to the blockage of fluorescence light from the choroid and choriocapillaris by the melanin of the RPE. After we remove the photoacoustic signal from the choroidal layer, 3D reconstruction was performed to obtain a 3D view of the retinal vasculature (see Video. 4). According to the image shown in Fig. 6f, both the retinal vasculature and network of fine neovascularization can be distinguished with PAM. This demonstrates that high-quality PAM, OCT, and FM of the RNV are both possible and improved with pigmented rabbits.

### Real-time B-scan for OCT and PAM

The real-time OCT B-scan and PAM B-scan results for normal rabbits and rabbits with RNV are shown in Videos 5, 6, 7 and 8. For a 7.2-mm B-scan line with 512 pixels, different layers of rabbit retina can be real-time displayed by OCT with a frame rate of 3.5 Hz. Motion artifacts caused by the rabbit’s heartbeat and breathing cannot be avoided in real-time results. The real-time PAM B-scan image was displayed with a 3.8-Hz frame rate and 256 pixels in a 7.2-mm B-scan line. Due to the selective absorption of hemoglobin, only the cross-section of retinal and choroidal blood vessel can be observed in the PAM B-scan imaging. The individual retinal blood vessel can be easily distinguished. As the density of choroidal vessels is higher in the choroidal layer than in the retinal layers, the choroidal layer looks more continuous than the retinal layers. For the rabbit with RNV, although the real-time OCT B-scan image can visualize different layers of the retina, it is difficult to distinguish the individual retinal blood vessel with the light blocked by proliferative preretinal membranes and hemorrhage above the retinal blood vessel layer. In contrast, the retinal blood vessel can still be distinguished clearly in the real-time PAM B-scan image.

### Image quantification

**Fig. 7:**
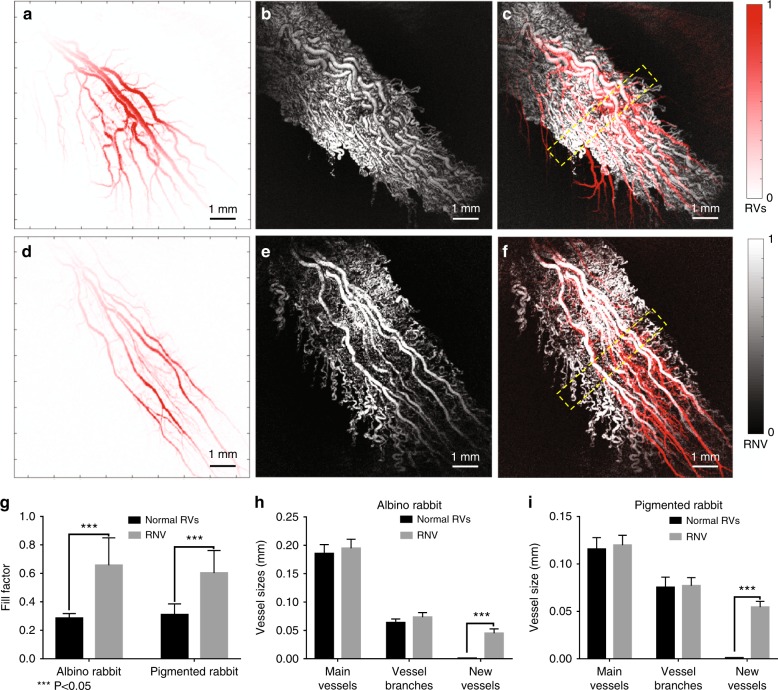
The comparison of PAM images between the normal retinal vessels and VEGF-induced RNV. **a** Normal retinal vessels in albino rabbit; **b** RNV induced by VEGF in albino rabbit; **c** composite pseudo color image of albino rabbit showing the retinal vessels in the same area before and after VEGF injection; **d** normal retinal vessels in pigmented rabbit; **e** RNV induced by VEGF injection in a pigmented rabbit; **f** composite pseudo color image of pigmented rabbit showing the retinal vessels in the same area before and after VEGF injection; **g** quantification of retinal vessels and RNV in albino and pigmented rabbits before and after VEGF injection using fill factor—statistical significance of the fill factor between a normal blood vessel and RNV (albino: *n* = 5; pigmented: *n* = 3; *P* < 0.05); **h** quantification of retinal vessels and RNV in albino rabbits before and after VEGF injection using the vessel size—statistical significance of the vessel size between a normal blood vessel and RNV (*P* < 0.05); **i** quantification of retinal vessels and RNV in pigmented rabbits before and after VEGF injection using the vessel size—statistical significance of the vessel size between a normal blood vessel and RNV (*P* < 0.05). (Pseudo color indicates the normal retinal vessels; grayscale shows the retinal vessels after injection with VEGF)

The PAM images acquired before and after VEGF injection are compared in Fig. 7 to further investigate the growth of RNV induced by VEGF. Points of correspondence between the two sets of images were determined from the PAM images with normal retinal vessels and neovascularization. Then, the two images were overlaid in different color bands as a composite pseudo color image. As shown in Fig. 7c, f, a significant increase in vascular tortuosity and numerous small new blood vessels can be observed after VEGF injection, which caused difficulty in image registration between the images acquired at different time points. Due to the high resolution of PAM and its alternative sensitivity to hemoglobin, PAM has the ability to quantify the RNV by extracting the retinal vessels from different layers and calculating the distribution of neovascularization in the region of interest (ROI). Rather than quantifying the RNV by using the area occupied by the leakage of fluorescein sodium on angiography, as is conventionally performed^27^, PAM can quantify neovascularization by using the number of pixels occupied by RNV at a pixel level, which is more accurate than the conventional fluorescein angiographic method, which is very dependent on the time after fluorescein instillation that images are acquired. The fill factor of the retinal vessels, which was defined as the percentage of retinal vessels in a special ROI, was applied to quantify the growth of RNV using following equation:

1 $${\it{{\rm R}}}_{\rm V} = \frac{{N_{{\rm RVs}}}}{{N_{{\rm ROI}}}}$$

where R_V_ indicates the fill factor of retinal vessels in the ROI, *N*_RVs_ presents the number of pixels occupied by retinal vessels, and *N*_ROI_ is the total number of pixels in the ROI. To improve the accuracy of quantification, the ROI of each imaging was set to 9 × 9 mm, which is smaller than the field of view of the transducer to avoid the out-of-focus issue.

The fill factors of normal retinal vessels (shown in Fig. 7a) and RNV (shown in Fig. 7b) in albino rabbits based on five albino rabbits were 22.46 and 66.11%, respectively. The fill factors in pigmented rabbits shown in Fig. 7d, e were 26.65 and 53.30% based on three pigmented rabbits. As shown in Fig. 7g a significant increase in fill factor (*P* < 0.05) can serve as a quantification of RNV and indicates the proportion of RNV in the same FOV. ImageJ was used to measure the vessel sizes of the main vessels, vessel branches, and neovascularization in the same area to further quantify the differences between normal retinal vessels and VEGF-induced RNV. To minimize the change in the vessel size at different positions, the width of the selected area was set to 1 mm, as indicated by the yellow dashed boxes in Fig. 7c, f. Three different locations (distal, middle, and proximal) of each vessel in the yellow dashed boxes were manually measured, and the measurements were averaged to minimize the measurement error. All the vessels in the yellow dashed boxes were involved in the statistical calculations. As shown in Fig. 7h, i, there is no significant difference in the vessel sizes of the main vessels and vessel branches before and after RNV induction. In contrast, RNV grew from nothing to a rich, dense vascular network (*P* < 0.05).

### Histology

**Fig. 8:**
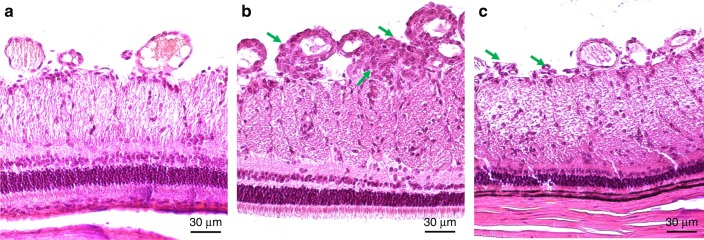
H&E stain histology results for albino and pigmented rabbits. **a** Normal retinal vasculature; **b** 7 days after VEGF injection to induce RNV; **c** 15 days after VEGF injection to induce RNV. (Green arrows indicate regions of RNV with an abnormal increase in vascular cells present on the inner retinal surface)

The hematoxylin and eosin (H&E) stain histology results shown in Fig. 8 were used to demonstrate the histologic structure of the irregular vascular network, which was visualized with the multimodality system. As demonstrated by the presence of vascular endothelial cells and the presence of red blood cells, the irregular small vessels were RNV induced by VEGF injections. Vascular cell nuclei on the inner retinal surface represent areas of RNV and are indicated with green arrows in Fig. 8. The histology results were consistent with the results from the multimodality imaging system.

## Discussion

This manuscript presents an integrated PAM, OCT, and FM evaluation of RNV in vivo in albino and pigmented large animal eyes. Compared with previous studies involving small animals, mostly focused on the evaluation of the anterior ocular vasculatures, the proposed multimodal system was uniquely designed for retinas of larger animal eyes, not only for structural imaging but also for functional and molecular imaging. Due to the conflict between the enlarged field of view and reducing the distortion of the laser beam, a unique telescope configuration, which combines a scan lens (SL) and an ophthalmic lens (OL), was specially designed according to the size of the human eye. A telecentric SL (LSM-03BB, Thorlabs, Newton, NJ), which will produce a flat image plane and a spot size that suffers minimal distortion, was utilized to achieve a constant spot size in the focal plane of the telescope configuration. A large field of view was achieved in the posterior ocular area via a 100-diopter OL (AC080–010-B-ML, Thorlabs, Newton, NJ). In addition, this system was designed to have high compatibility with multiple wavelengths for functional PAM imaging and molecular imaging utilizing different dyes through the sharing of a tunable optical parametric oscillator (OPO) laser system for both PAM and FM imaging.

Multimodality imaging provides unique advantages to visualize the anatomy and pathophysiology of diseases. OCT creates an image based on low-coherence interferometry and analyzes the difference in back-scattered light, comparing the tissue to a reference arm. OCT B-scan imaging allows for excellent visualization of the different retinal layers. Due to the preretinal fibrovascular tissue above the retinal vasculature, OCT has difficulty in distinguishing normal vasculature and neovascularization even with the difference in the blood vessel wall between normal retinal vessels and neovascularization. Unlike optical scattering-based OCT imaging, PAM imaging is based on the optical absorption properties of tissue. Melanin and hemoglobin are the two primary endogenous absorbers in the eye. Working within the optical spectrum sensitive to hemoglobin, PAM can be used to image not only blood vessels, but also bleeding. Although the vessel size and blood flow in neovascularization are smaller than those of normal vessels, PAM is still sufficiently sensitive to detect neovascularization and the network of small, irregular vessels. By comparing the images acquired before and after RNV induction, RNV can be visualized clearly. The fill factor is proposed to quantify the RNV in an ROI with pixel-level resolution and accuracy, which is uniquely possible with PAM. Importantly, this study demonstrates that whereas melanin results in a strong PAM signal, the high-resolution nature of this system allows for visualization of neovascularization even in pigmented rabbits. In this multimodality system, FM can also be performed using intravenous injection of fluorescein. High contrast is achieved with FM in pigmented rabbits, where melanin in the RPE blocks the light from the choroid and choriocapillaris vasculature. In RNV, significant progressive hyperfluorescence is noted, indicating the leakage of fluorescein dye due to RNV. The FM images of albino rabbits suffer from diffuse hyperfluorescence, limiting visualization of the RNV, and FM in pigmented rabbits exhibits improved visualization of retinal vasculature and RNV. Multimodality imaging, which can combine the merits and compensate the limitations of each modality to give additional information that cannot be gleaned from a single modality, will be very beneficial in the field of ophthalmology.

This study combines PAM, OCT, and FM and presents high-quality visualization of the retinal vasculature and neovascularization in albino and pigmented rabbits in vivo. This is the first study to perform multimodal imaging, particularly PAM imaging, of RNV in rabbit eyes, and it demonstrates that high-quality, high-resolution images can be achieved below the ANSI safety limit even with the presence of melanin. The current study is also the first that has involved a neovascularization disease model of the retina in large animal eyes with multimodality high-resolution PAM, OCT, and FM imaging and demonstrates in 3D the spatial distribution of the retinal vasculature and neovascularization. As the PAM signal is attenuated with distance from the detector, the rabbit axial length of 18.1 mm, which is similar to that of humans, three times greater than that of rats and six times greater than that of mice, can pose a challenge for PAM of rabbit eyes. Thus, this work presents a significant step in the clinical translation of this technology. The success achieved in this work demonstrates that PAM could play an important role in the diagnosis of RNV-related diseases, such as diabetic retinopathy, sickle cell retinopathy, retinal vein occlusions, and retinopathy of prematurity. Unlike the motion contrast imaging involved in OCTA, PAM images blood vessels based on the strong optical absorption of hemoglobin. Thus, the blood flow velocity is not a limitation in PAM angiographic imaging. Blood vessels with various sizes, even angiogenic microvessels with very slow flow, and local bleeding where the blood is not flowing can all be imaged by PAM with high sensitivity. Moreover, PAM has a greater depth of penetration than other optical imaging techniques, such as OCT, and can thus provide improved visualization of the deeper layers, such as the choroid that OCT has difficulty imaging. In addition, PAM is superior in functional imaging, including quantitative evaluation of blood velocity and blood oxygen saturation, as demonstrated in previous studies^28^, which is highly valuable for ophthalmology applications. The addition of exogenous optically absorbing molecules, such as gold nanoparticles, iron oxide, carbon nanomaterials, and targeted molecular probes, can be used to perform multimodal molecular imaging.

In summary, an integrated multimodality imaging system, which combines OCT, PAM, and FM, is described, and the first quantified PAM images of RNV are demonstrated in large animal eyes in vivo using VEGF-induced RNV. Both albino and pigmented rabbits yield high-quality images, and melanin results in improved image quality. This indicates the universal adaptability of our system. The results demonstrate that this multimodality imaging system can non-invasively visualize RNV in larger animal eyes using OCT and PAM and verify the leakage of neovascularization using FM and fluorescein dye. This model of RNV closely mimics several of the leading causes of blindness, including proliferative diabetic retinopathy. Safe and high-resolution imaging achieved on rabbit eyes that have similar sizes to human eyes paves the road to clinical translation of the technology.

## Materials and methods

### Experimental system

A schematic diagram of the multimodality retinal and choroidal imaging system with integrated spectral-domain OCT (SD-OCT), PAM, and FM is shown in Fig. 1.

The details regarding our PAM modality and OCT modality can be found in our previous papers^23,24^. In brief, SD-OCT was adapted from a commercially available OCT system (Ganymede-II-HR, Thorlabs, Newton, NJ). An OPO laser (NT-242, Ekspla, Vilnius, Lithuania) was used as the illumination source for PAM and FM with a repetition rate of 1 kHz. The laser light was first collimated by a spatial filter, reflected by a dielectric mirror and a dichroic mirror (DM), and then combined with the OCT light. Sharing the same galvanometer, the PAM laser light and OCT laser light were delivered and focused on the same area of the rabbit retina through a telescope configuration. A custom-built needle-shaped ultrasound transducer with a central frequency of 27.0 MHz (Optosonic Inc., Arcadia, CA, USA) ^29^ was placed in contact with the sclera and coupled with balanced salt solution (BSS) to detect the photoacoustic signal. The photoacoustic signal was amplified by a 57-dB low-noise amplifier (AU-1647, L3 Narda-MITEQ, NY) and digitized with a sampling rate of 500 MHz. Simultaneously, the OPO laser output energy for PAM illumination was acquired by a photodiode (PD) and digitized using the same DAQ Card with the same sampling rate (PX1500–4, Signatec Inc, Newport Beach, CA).

The FM system consists of a DM, an appropriately selected filter, and an avalanche photodiode (APD). The incident light for FM is the same as for PAM: the OPO laser. The incident light has the same optical path as for PAM and OCT. The FM will thus focus on the same area as PAM and OCT. The fluorescence light emitted travels back to the telescope configuration and galvanometer and is reflected by a dichroic mirror and detected by the APD after passing through the appropriate filter. The signal from the APD was digitized using the same DAQ card as PAM.

The OPO laser, the OCT scan header, and the DAQ card were synchronized through a multifunction I/O device (USB-6353, National Instruments Corporation, Austin, TX). A delay generator and a clock switch circuit were used to select the system clock between the OPO and OCT systems.

The light from different imaging modalities was coaxially aligned to ensure co-registration of the multimodality images. The lateral resolutions of PAM and SD-OCT were previously quantified to be 4.1 and 3.8 μm, respectively; whereas, the quantified axial resolutions of PAM and OCT were 37.0 μm and 4.0 μm, respectively^23,24^. In this study, a laser wavelength of 905 nm and a laser energy of 1.25 mW in front of the cornea were applied for OCT, a laser wavelength of 532 nm and a laser energy of 80 nJ per pulse before the eye were used for PAM, and a laser wavelength 480 nm and a laser energy of 2 nJ per pulse were utilized for FM. According to the ANSI eye safety limit, the laser energy used for PAM and FM should not exceed 146 nJ, whereas for the 905 nm CW laser used for OCT, the laser energy should be less than 1 mW. The detailed procedure for the safety calculation can be found in supplemental information and has been discussed in our previous work^23,24^. According to our simulation, the laser energy used in PAM is less than 60% of the ANSI safety limit. Although our OCT light exceeded the ANSI safety limit, it can be easily reduced by using one of many available clinical OCT systems, which have lower laser energy and higher image quality. Fifty-degree fundus photography was performed (TRC-50EX, Topcon Corporation, Tokyo, Japan) along with fluorescein angiography using a commercially available human clinical system. Subsequently, the multimodality imaging system was used to acquire OCT, PAM, and FM images.

### Experimental parameters and procedures

In OCT mode, the system employed a 5.5-kHz clock from a computer. For each B-scan, the scan line contained 512 pixels in 10.5 mm and was an average of 3 scans; the frame rate was 3.5 Hz, and the resolution was 20.5 μm. Both the PAM mode and FM mode shared the same laser system with a 1-kHz laser clock. The scan sizes of PAM and FM were 512 × 512 and 256 × 256, respectively. With a scan range of 10.5 mm × 10.5 mm, the corresponding resolutions were 20.5 μm and 41 μm, respectively.

### Animal preparation

All the experimental procedures were performed in accordance with the ARVO (The Association for Research in Vision and Ophthalmology) Statement for the Use of Animals in Ophthalmic and Vision Research and were approved by the Institutional Animal Care & Use Committee (IACUC) of the University of Michigan (Protocol PRO00006486, Photoacoustic & Molecular Imaging of the Eye, PI: Paulus).

Nine albino rabbits (New Zealand White rabbits) and five pigmented rabbits (Dutch-Belted rabbits) were involved in this study. The retinal vessels of both the albino rabbits and pigmented rabbits were imaged in vivo. The animals were anesthetized with a mixture of ketamine (40 mg kg^−1^) and xylazine (5 mg kg^−1^) via intramuscular (IM) injection. Following this, anesthesia was maintained with 2% isoflurane and oxygen using a V-Gel® (D10004, Jorgensen Laboratories, Loveland, CO). Before the experiment, subcutaneous injection with one dose of meloxicam was administered to the rabbit. The pupils of the eyes were dilated with topical application of phenylephrine hydrochloride 2.5% and tropicamide 1% eye drops. Topical tetracaine drops were applied for additional topical anesthesia prior to initiation of the experiments. To evaluate anesthesia levels and temperature, continuous monitoring of the heart rate and respiratory rate was performed using a V8400D Capnograph & SpO2 Digital Pulse Oximetry (MWI Animal Health, Boise, ID). Rectal temperature was measured every 15 min and used to adjust a water-circulating heating pad (TP-700, Stryker Corporation, Kalamazoo, MI) to keep the body temperature stable. BSS (Altaire Pharmaceuticals, Inc., Aquebogue, NY) was applied to wash the cornea to keep it moist and couple the ultrasound transducer to the sclera. For the FM imaging, fluorescein sodium (10%, 0.1 mL kg^−1^, Akorn Inc, Lake Forest, IL) was injected through the marginal ear vein.

### RNV model

RNV was achieved through intravitreous injection of VEGF (Human VEGF-165, Shenandoah Biotechnology Inc, Warwick, PA) to induce RNV in the albino and pigmented rabbits^30,31^. The eyelid was retracted with a pediatric Barraquer wire speculum, and the conjunctiva was grasped with forceps to ensure stability. The conjunctiva was cleaned with 5% povidone-iodine, and an intravitreous injection was performed using a 30 G ½ inch needle inserted 3 mm posterior to the limbus through the pars plana. The needle was directed at an angle of ~45 degrees to avoid touching the rabbit lens. A total of 0.1 mL of VEGF-165 (100 µg mL^−1^) was injected into the vitreous cavity. The conjunctiva was then irrigated with BSS, and the speculum was then removed. The animal was followed serially after induction of RNV with intravitreous injection.

### Positioning of multimodality imaging and histology

**Fig. 9:**
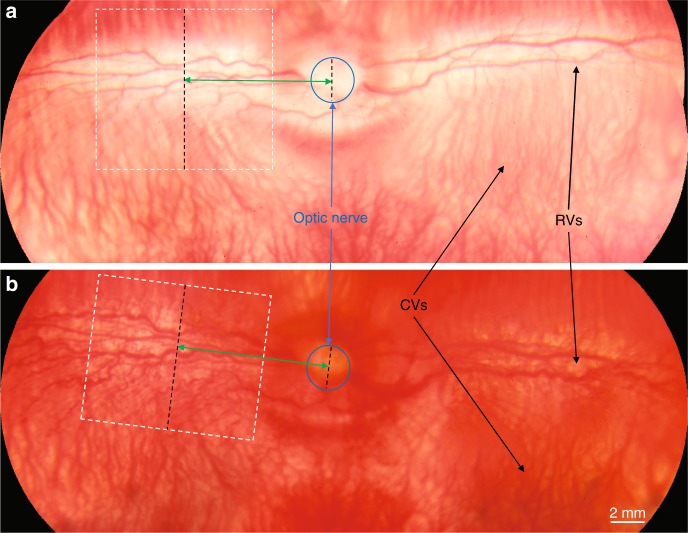
Schematic of positioning in multimodality imaging and histology sectioning. **a** Color fundus of rabbit retina before VEGF injection; **b** color fundus of rabbit retina after VEGF injection. (White dashed boxes indicate the region of interest in multimodality imaging, blue circles show the position of the optic nerve, and green arrows present the distance between the region of interest and optic nerve along the retinal vessels)

The normal retina of the rabbit before VEGF injection is shown in Fig. 9a, where color fundus photography demonstrates a very wide field of view of the rabbit retina. After VEGF injection, the growth of RNV in the whole retina is shown in Fig. 9b. The color fundus images taken in advance were used to determine the ROI for the multimodality imaging. The optic nerve, which is marked with a blue circle, can be used as both a main landmark and a ruler (given its stable size) in multimodality imaging and histology analysis. We can calculate the distance (marked as green arrows) between the optic nerve and the ROI (marked as white dashed boxes) in multimodality imaging. After the eye is removed for histology analysis, the retinal blood vessels and optic nerve in the in vitro tissue can be used to locate the ROI in imaging experiments. Slides can be sectioned from a corresponding area to ensure that the area of histology analysis is consistent with the one for the imaging.

## Electronic supplementary material

Supplemental Information

Video 1

Video 2

Video 3

Video 4

Video 5

Video 6

Video 7

Video 8
