# Supplementary material for: High-resolution, in vivo multimodal photoacoustic microscopy, optical coherence tomography, and fluorescence microscopy imaging of rabbit retinal neovascularization
Source: Light Sci Appl. 2018 Dec 5;7:103. doi: 10.1038/s41377-018-0093-y (PMC6281580; doi:10.1038/s41377-018-0093-y)
Supplement: Supplementary file 1 — Supplemental Information [file 41377_2018_93_MOESM1_ESM.docx]

**Supplemental Information**

**High-resolution, *in vivo* Multimodality Photoacoustic Microscopy, Optical Coherence Tomography, and Fluorescence Microscopy Imaging of Rabbit Retinal Neovascularization**

Wei Zhang^1,2^, Yanxiu Li^3,4^, Van Phuc Nguyen^3^, Ziyi Huang^1^, Zhipeng Liu^2^†, Xueding Wang^1,5^†, Yannis M. Paulus^1,3^0F†

^1^Department of Biomedical Engineering, University of Michigan, Ann Arbor, MI 48105, USA;

^2^Institution of Biomedical Engineering, Chinese Academy of Medical Science & Peking Union Medical College, Tianjin, 300192, China;

^3^Department of Ophthalmology and Visual Sciences, University of Michigan, Ann Arbor, MI 48105, USA;

^4^Department of Ophthalmology, Xiangya Hospital, Central South University, Changsha, Hunan, 410008, China;

^5^Department of Radiology, University of Michigan, Ann Arbor, MI 48105, USA

**Experimental Setup**

The optical part of the system setup has been described in the experimental system and Fig. 1a. As we describe in the manuscript, three different modalities are sharing the same galvanometer, shown in Fig. 1Sa. Since the CW SLD light for OCT and OPO laser for PAM and FM are combined together before the scan head, the three different modalities have the same maximum scan range. The diameter of maximum scan range after the scan head $D_{S}$ is 1 cm, the corresponding scan range inside the eye $D_{E}$ is

$D_{E}=\frac{f_{e}}{f_{o}}\times D_{S}=17.9 mm$ (1)

where $f_{o}=10 mm$ is the focal length of the ophthalmic lens, $f_{e}=17.9 mm$ is the focal length of the optical system in rabbit eye.

The scan path of the laser spot in the region of interest is shown in Fig. 1Sb. In OCT mode, the system is working at 5.5 kHz by the clock from the computer. For each B-scan, the scan line contains 512 pixels in 10.5 mm and 3 averages, therefore the frame rate is 3.5 Hz. In PAM mode, with a 1 kHz laser clock, the frame rate is 3.8 Hz for B-scan with 256 pixels in a scan line. For a given 512×512 pixels volume scan, the galvanometer controls the scan spot to scan each B-scan in same direction, shown in blue arrows. The laser spot will move from the end of each B-scan line to the start of next B-scan, shown in black dash arrows. The change of positions between two B-scan lines takes 11 triggers. For a given PAM volume scan with 512×512 pixels, it takes $\left( 512+11 \right)\times\frac{512}{1000}=268 s$; while, the given FM scan with 256×256 pixels takes 68 s.

For the penetration depth, OCT is limited by the system of Ganymede-II-HR and the control software of ThorImage with 1.89 mm. For PAM imaging, it is only limited by the penetration of incident light, which is about 1mm here with 532 nm laser. FM is a 2D imaging modality and does not include image depth information.


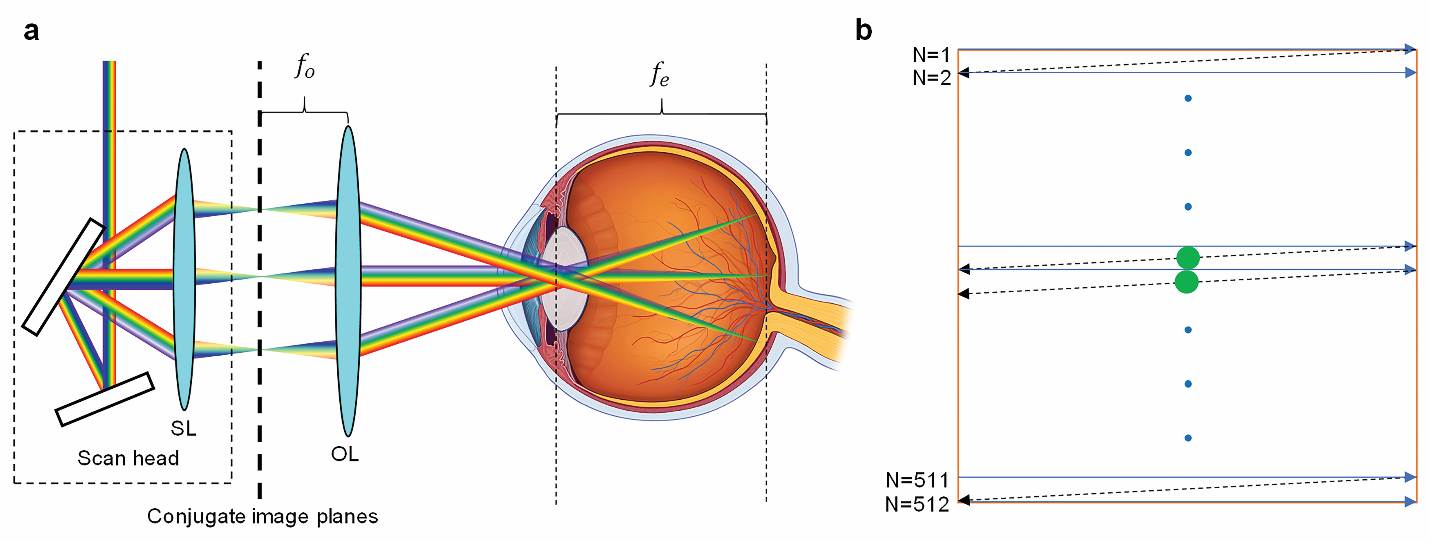


Figure 1S. Scanning part of the system and the scan path of the laser spot in the region of interest (ROI). (a) Scanning part of the system;(b) scanning path during the imaging. (Blue arrows show the path of each B-scan; dashed arrows indicate the path of laser spot from one B-scan to next B-scan.)

**Laser Safety**

Laser safety is an incredibly important aspect in ocular imaging. In this study, the OCT light source is a combination of two individual superluminescent diodes, one centered at 846 nm and the other one at 932 nm, for a central wavelength of 905 nm and an estimated bandwidth of about 220 nm with a laser energy of 1.25 mW on the cornea for OCT imaging; the laser wavelength of 532 nm and the laser energy of 80 nJ per pulse before the eye were used for PAM; while, the laser wavelength 480 nm and laser energy of 2 nJ per pulse were utilized for FM. The OCT light source used in this study is higher than ANSI safety limit, which is 1 mW^1^. Since the current OCT system is not designed for ocular imaging, it would be easy to reduce the OCT laser energy by using any number of currently available clinical OCT systems.

As shown in Figure 3b and Figure 4b, the minimum detectable retinal blood vessel in PAM images is 20.5 μm, which is the same as the given resolution of imaging setup. Since the laser light was collimated before cornea, the corresponding laser spot in the retina areas should be around 20-25 μm^1,2^. The angular subtense of the source can be calculated by following equation^3^:

$\alpha=\frac{a}{f}=\frac{25\mu m}{17mm}=1.47 mrad<\alpha_{min}=1.5 mrad$ (2)

where α is the laser spot in the retina and $f$ is the focal length of the human eye. The laser spot is estimated to be 25 μm by more conservative calculations. Following the threshold of the angular subtense of the apparent source, the light source in this study can be regarded as a small (point) source. For potential clinical translation, three different ANSI safety limits need to be calculated to evaluate the safety of this system^4^. Similar tests with our current system have been calculated in our previous publications^5,6^.

Rule 1: Single pulse maximum permissible exposure (MPE). For small source Nano-second laser (400 nm-700 nm), the MPE should be considered as followed:

${MPE}_{SP}=5.0\times{10}^{-7} J/{cm}^{2}$ (3)

Rule 2: Average power MPE for thermal and photochemical hazard. For a given $512\times512$ scan area with 11 points fly time per B-Scan line, the totally laser exposure time $T_{max}$ is around 268 s with the corresponding ${MPE}_{total}$.

${MPE}_{total}=T_{max}\cdot1\times{10}^{-3}J/{{cm}^{2}}=2.68\times{10}^{-1}J/{{cm}^{2}}$ (4)

As shown in the scan pattern in Figure 1S A, each individual B-scan in a given volume scan is from left to right. It takes 11 triggers for the galvanometer to move the scan spot from the end of a B-scan line to the start of next B-scan line, whose motion path is shown by the dash back arrow. With the given resolution of imaging and maximum laser spot with collimated light, the distance between two motion paths should be around 20.5 μm, which is smaller than the maximum laser spot. The maximum pulse number that any given area will have during a 268 s laser exposure $n_{max}$is $3$. The minimum MPE per pulse of each individual area within a volume scan is

$\frac{{MPE}_{total}}{n_{max}}=\frac{2.68\times{10}^{-1}}{3}J/{{cm}^{2}}={8.9\times{10}^{-2}J}/{{cm}^{2}}$ (5)

Rule 3：Multiple-pulse MPE for thermal hazards. The multiple-pulse MPE is calculated from signal pulse MPE with a multiple-pulse correction factor.

${MPE}_{MP}=n_{max}^{-0.25}\cdot{MPE}_{sp}=3^{-0.25}\times5.0\times{10}^{-7} J/{cm}^{2}=3.8\times{10}^{-7} J/{cm}^{2}$ (6)

As rule 3 will give the most conservative value, the maximum permissible single laser pulse energy in current setup is shown below with a *D* = 7 mm given the pupil diameter of a human^1^.

$E={MPE}_{MP}\cdot\pi\cdot\left( \frac{D}{2} \right)^{2}=146 nJ$ (7)

The laser energies involved in the PAM and FM studies, which are 80 nJ and 2 nJ per pulse, are significantly lower than the ANSI safety limit. While additional safety tests after both single and repeat PAM imaging are warranted prior to translating PAM into clinical use including electroretinography (ERG), optokinetic visual acuity, histopathology, electron microscopy, and immunohistochemistry including TUNEL assay and other tests for inflammation, cell injury, and cell death in both the short and long term, the current study still demonstrates the potentially safe application of PAM in ocular angiogenesis.

**Data Processing**

In OCT mode, the images were directly acquired by the control software (ThorImage, Thorlabs, Newton, NJ) of Ganymede-II-HR. For this commercial system, no data processing was involved in OCT imaging. For our laser scanning PAM system, the data for volume scan was acquired by each A-scan. Since the diameter of the laser beam targeted to the retina and thickness of the retinal layer is much smaller than the region of interest, the scattering of light was ignored in data processing. A digital high pass filter with 10 kHz cutoff frequency was used to remove the DC and low-frequency background drift of each A-scan signals, and a low pass filter with cutoff frequency at 100 MHz was applied to remove the high-frequency noise. All the A-scan signals were further normalized by recorded pulse by pulse energy. Deconvolution was applied to each A-scan signal with the response function of the transducer to obtain the distribution of optical absorption. All the A-scan data was combined to form a 3D image. Since the distance between the retinal blood vessel layer and the choroidal blood vessel layer is more than 200 μm, depth-based segmentation can be used to extract the retinal blood vessel layer. Peak value projection was applied to form a 2D imaging from 3D data. A more uniform 2D image is obtained with the compensation of the acoustic field of the transducer. Due to the high SNR of fluorescence imaging, the peak value projection was directly applied to form a FM image.

**Reference**

1. American National Standards Institute. American national standard for safe use of lasers. *Laser Institute of America* 2007.
2. Schulmeister K, Althaus SJ, Grabner U, Vees G. Location and size of the apparent source for laser and optical radiation ocular hazard evaluation.2004.
3. Henderson A. Guide to Laser Safety. *Springer Science & Business Media* 1997.
4. Hu S, Rao B, Maslov K, Wang LV. Label-free photoacoustic ophthalmic angiography. *Opt Lett* 2010; **35**: 1-3.
5. Tian C, Zhang W, Mordovanakis A, Wang XD, Paulus YM. Noninvasive chorioretinal imaging in living rabbits using integrated photoacoustic microscopy and optical coherence tomography. *Opt express* 2017; **25**: 15947-15955.
6. Tian C, Zhang W, Nguyen VP, Wang XD, Paulus YM. Novel Photoacoustic Microscopy and Optical Coherence Tomography Dual-modality Chorioretinal Imaging in Living Rabbit Eyes. *J Vis Exp* 2018; **132**: doi: 10.3791/57135.
